# The effect of adding ethanolic saffron petal extract and vitamin E on growth performance, blood metabolites and antioxidant status in Baluchi male lambs

**DOI:** 10.5713/ajas.18.0615

**Published:** 2019-02-14

**Authors:** Fereshteh Alipour, Alireza Vakili, Mohsen Danesh Mesgaran, Hadi Ebrahimi

**Affiliations:** 1 Department of Animal Science, Faculty of Agriculture, Ferdowsi University of Mashhad, Mashhad 9177948974, Iran

**Keywords:** Antioxidant Status, Baluchi Lambs, Malondialdehyde, Muscle Tissue, Saffron Petal, Vitamin E

## Abstract

**Objective:**

This study investigated the effects of the administration of ethanolic saffron petal extract (SPE) and vitamin E (Vit E) on growth performance, blood metabolites and antioxidant status in Baluchi lambs.

**Methods:**

Thirty-two Baluchi male lambs (35.22±5.75 kg) were randomly divided into 4 groups. The 1st (control), 2nd (injectable saffron petal extract [ISPE]), and 3rd (Vit E) groups were respectively injected subcutaneously with either physiological saline (5 mL), SPE (25 mg/kg body weight [BW]) or DL-α-tocopheryl acetate (225 IU) once a week. An oral dose of SPE (500 mg/kg BW) was also administered to the 4th group (oral saffron petal extract [OSPE]). Feed intake and BW were measured for 42 days and blood samples were taken on days 1, 14, 28, and 42. The lambs were slaughtered, and tissue samples were taken.

**Results:**

Growth performance and many blood metabolites were not affected (p>0.05) by the treatments. Cholesterol of plasma in the ISPE and Vit E groups was similar and less (p< 0.01) than both the OSPE and control groups. Although there was no significant difference between the control and other groups for plasma triglyceride, the ISPE group showed lower (p<0.05) triglyceride than the OSPE and Vit E groups. The highest (p<0.01) plasma glutathione peroxidase (GPx) was detected in the OSPE group, while the ISPE and Vit E groups showed higher (p<0.01) superoxide dismutase (SOD) of plasma than the control. Malondialdehyde of plasma in the ISPE group was lower (p<0.05) than the OSPE. No differences (p>0.05) were observed among the groups for antioxidant status of both longissimus dorsi muscle and liver. However, the activity of GPx in the kidney and heart, as well as SOD activity in the kidney, were influenced (p≤0.01) by the treatments.

**Conclusion:**

Adding ethanolic SPE improved antioxidant status and lowered lipids oxidation in lambs. The SPE and Vit E demonstrated similar effects on antioxidant status in lambs.

## INTRODUCTION

Lipids oxidation decreases the quality and shelf-life of frozen and processed meat [1]. Free radicals like reactive nitrogen and oxygen species (RNS and ROS), which are formed and destroyed by aerobic organisms, are the reason for oxidative damage, with effects on many biological molecules such as lipids, proteins, carbohydrates and nucleic acid [2]. Antioxidant protective systems, including enzymatic (catalase, glutathione peroxidase [GPx], and superoxide dismutase [SOD]) and non-enzymatic (A, C, E vitamins [Vit], peptides of glutathione, cofactors (Q_10_), zinc and selenium minerals, phenolic acids and nitrogen compounds of uric acid), play an important role in modulating oxidative stress-induced cell injuries by scavenging free radicals [3]. The non-enzymatic antioxidants can be classified into two major categories based on their source: synthetic and natural antioxidants [3]. Synthetic antioxidants, such as butylated hydroxytoluene, butylated hydroxyanisole, and tertiary butylhydroquinone, are commonly used in the food industry. However, the health risks associated with consuming synthetic antioxidants have led to a growing interest in the use of natural alternatives [4].

There is scientific evidence that the quality and shelf-life of the meat may improve by pre-slaughter dietary supplementation, or by post-slaughter treatment of the carcass with antioxidants from natural sources [5]. Plants’ secondary metabolites, especially phenolic compounds and some of their derivatives, are highly effective in preventing oxidation because of the significant relationship between the antioxidant activity and the phenolic compounds [6].

*Crocus sativus* L., commonly known as saffron, is a perennial plant of the Iridaceae family that is widely cultivated in Iran as it is well adapted to arid and semi-arid lands. Saffron has long been used in medicine and foods as a condiment or when trying to give it a yellow colour. Saffron petal, as an important by-product of saffron, is produced in large amounts annually (more than 10,000 tons/yr) and usually discarded as a waste product [7]. The reported antioxidant properties of saffron petal [8,9] are more likely attributed to its phenolic compounds, such as crocin and kaempfrol [10]. However, there are limited studies on the effects of saffron petal, and/or its bio-active compounds, as an antioxidant source for ruminant animals. Therefore, the purpose of this research was to investigate the effects of saffron petal extract on the growth performance, as well as on the plasma and tissue antioxidant status, of lambs.

## MATERIALS AND METHODS

### Extract preparation

Saffron petals were collected from the Bakherz district in Khorasan Razavi province in the north-east of Iran in November 2016. A voucher specimen (No. 44557) of the plant was identified in the Herbarium of Ferdowsi University of Mashhad. The petals were pulverized using a grinder after being shade-dried. The ethanolic saffron petal extract (SPE) was prepared by dissolving 50 g of the dried petal powder in 1,000 mL of ethanol (80% v/v) and shaking it for 72 h (GFL Orbital Shaker 3005, Burgwedel, Germany) at room temperature. Then, the extract was filtered through a Whatman No. 1 paper (Whatman Ltd., Maidstone, England). The residual solvent of the ethanolic extract was removed under reduced pressure at 38°C using a rotary evaporator (Heidolph Laborota 4000, Schwabach, Germany). The condensate extract was completely dried using a freeze-drying system (Martin Cherist, Beta 2-8 LD plus, Osterode am Harz, Germany). The final powdered extract was then weighed to calculate the ethanolic SPE yield (w/w), which was 42%. The extract powder was stored in dark bottles at 4°C until use [11]. Finally, the extract was dissolved in normal saline for injections.

### Total phenolic and flavonoid compounds determination

The total phenolic content was determined using the Folin-Ciocalteu method [12]. Briefly, a three-fold serial dilution of gallic acid (0.02, 0.05, and 0.1 mg/mL), as well as SPE solution, were prepared in distilled water to final volume of 0.5 mL. Then 0.25 and 1.25 mL of Folin-Ciocalteu reagent (1 N) and sodium carbonate solution (20%), respectively, were added in test tubes. Afterwards, the tubes were vortexed and the absorbance was recorded at the wavelength of 725 nm after 40 min incubation at room temperature. Finally, the total phenol content of the extract was calculated using a standard curve and reported as mg of gallic acid equivalents (GAE) per gram of the extract.

Total concentrations of flavonoids were measured by the aluminum chloride colorimetric method [13]. To this aim, 0.5 mL of the extract (before drying) was dissolved in 1.5 mL methanol, followed by adding 0.1 mL of aluminum chloride 10% and 0.1 mL of 1 M potassium acetate solution. Finally, 8.2 mL of distilled water was added, and the compound was stored at room temperature for 30 min. Absorbance of the reaction mixture was measured at 415 nm against a methanol blank. The concentration of flavonoid was reported as mg of quercetin equivalents (QE) per gram of the extract.

### High performance liquid chromatography

Determination of flavonoids in SPE was performed by the high performance liquid chromatography method using a liquid chromatography apparatus equipped with a 1000 Smartline pump, a 5000 Smartline manager solvent organizer, a 250×4.6 mm Eurospher 100-5 C18 analytical column, and a 2800 Smartline photo-diode array (Knauer, Berlin, Germany) as descripted by Mattila et al [14]. Samples were filtered through a syringe filter (PTFE, pore size 0.22) before injection. Twelve microlitres of the sample were injected through a 3900 Smartline Auto-sampler injector equipped with a 100 μL loop, and the temperature of the column was controlled at 25°C using a Jet Stream 2 Plus oven (Knauer, advanced scientific instrument, Germany). Methanol (0.05% trifluoroacetic acid [TFA]) and water (0.05% TFA) were employed as a mobile phase with a flow rate of 0.5 mL/min. The peaks were monitored at 320 nm and data acquisition and integration were performed using EZChrom Elite software (Darmstadt, Germany).

### Guidelines for dosage calculation

The injectable dosage of SPE was calculated using the model of the US Food and Drug Administration (USFDA) [15]. This model has been developed to calculate the human equivalent dose of a drug, or medicinal herbals, and can be used in other mammalians as well [15]. According to the USFDA model, the injectable dosage of SPE was calculated on the basis of body weight (BW) and body surface area as follows:

Dosage for sheep (mg/kg BW)=dosage for rat (mg/kg BW)×rat Km/sheep Km

where dosage for rat is 150 mg/kg BW [16], rat km is 6 [17], sheep km is a ration of BW (kg) to body surface area (m^2^) [15]. According to Mitchell’s model [18], the body surface area is calculated as 0.09×BW^0.67^.

### Animals and experimental design

This study was undertaken from July to September of 2016 at the Experimental Farm of the Department of Animal Sciences, Ferdowsi University of Mashhad. Thirty-two Baluchi male lambs aged 4 to 6 months, with the mean initial BW of 35.22±5.75 kg, were used in a 56-day trial. After performing random stratification on the basis of BW, the lambs were divided into 4 groups (n = 8) and housed in individual pens. Once a week, the first (control) and second group (ISPE) received a subcutaneous injection of a 5 mL physiological saline and 25 mg/kg BW SPE, respectively. The 3rd (Vit E) group received a subcutaneous injection of 225 IU of DL-α-tocopherol acetate (a total dose of 1,350 IU) once a week. The 4th group (OSPE) received an oral SPE dose equivalent to 500 mg/kg of BW in a liquid form once a week. It should be noted that DL-α-tocopherol acetate was included in the experiment as a positive control. The treatments were applied for 6 consecutive weeks. All the lambs were fed a basal diet (Table 1) twice a day at 08:00 and 17:00. The basal diet was formulated using Small Ruminant Nutrition System, a mathematical model based on the Cornell Net Carbohydrate and Protein System [19], to meet the lambs’ requirements. The chemical composition of the diet was determined using standard methods [20,21]. *Ad libitum* water was also available. The lambs were adapted to the diets and pens for 14 days. They were treated against internal and external parasites, with 10 mg/kg of albendazole and 0.22 mg/kg of ivermectin, 30 days before initiation of the experiment.

### Growth performance

Weight of feed offered and ort were recorded daily and dry matter intake (DMI) was computed as average daily DMI. The lambs were weighed weekly before the morning feed. Initial and final BW, average daily gains (ADG), body weight gains (BWG), and feed conversion ratios (FCR) were also measured.

### Blood sampling and analysis

On the 1st, 14th, 28th, and 42nd days of the trial period, the blood samples were collected from the jugular vein of all lambs before the morning feed [22]. Two blood samples (10 mL) were taken into vacutainer tubes, one containing sodium heparin for plasma separation, and one containing K_3_- ethylenediaminetetraacetic acid for whole blood analysis. After collection, the samples were held on ice for approximately 3 h. The plasma was obtained by centrifugation at 3,000 rpm at 4°C for 10 min and then frozen at −20°C until analysis, while whole blood samples were processed immediately.

The plasma samples were analyzed for albumin, total protein (TP), alanine aminotransferase (ALT), aspartate aminotransferase (AST), creatinine, triglyceride (TG), cholesterol and glucose levels using commercially available kits (Pars Azmoon Laboratory, Tehran, Iran) and an Autoanalyzer (Biotecnica, Targa 3000, Rome, Italy).

White blood cell (WBC) and red blood cell counts, as well as platelets, mean cell volume, packed cell volume, haemoglobin level, and mean cell haemoglobin concentration were determined in whole blood samples using a cell counter (Nihon Kohden Celltac alpha MEK-6450 haemotology analyzers, Tokyo, Japan). An optical microscope (10×), with the smear slides stained with Giemsa, was employed to obtain the differential counts of WBCs, including lymphocyte, monocyte, neutrophil, and eosinophil [22].

### Slaughter surveys and tissue sampling

At the end of the trial period, the lambs were fasted overnight with free access to water and then slaughtered in a semi-industrial abattoir. Hot carcass weight was recorded and the carcasses were kept at 4°C for 24 h to measure cold weight. The left side of the longissimus dorsi muscle between the 12th and 13th ribs, as well as the liver, kidney and heart tissues, were sampled immediately after slaughter, frozen in liquid nitrogen, wrapped in aluminum foil and kept at −80°C until further analysis [23].

### Antioxidant status evaluation

The plasma GPx activity was determined using a Ransel kit (Randox Laboratories Ltd., 55 Diamond Road, Crumlin, County Antrim, UK, BT29 4QY) according to the method of Paglia and Valentine [24]. The SOD and total antioxidant capacity (TAC) in plasma were measured using a Ransod kit [25] and a Randox kit [26], respectively. The level of malondialdehyde (MDA) was also measured using thiobarbituric acid-reactive substances in accordance with the method of Kaya et al [27]. To measure the antioxidant statuses of the tissues, the longissimus dorsi muscle, liver, kidney and heart samples, were homogenized in 1.15% KCl solution and then analyzed according to the methods described above.

### Statistical analysis

The data were analyzed using the general linear model procedure of SAS (SAS 9.1; SAS Inst. Inc., Cary, NC, USA) as a completely randomized design. The means were compared using Tukey’s test.

## RESULTS

### Phenolic and flavonoid compounds

The total phenolic and flavonoid content, as well as some types of phenolic compounds in the SPE, is listed in Table 2. The extracted powder of saffron petal contained 4.29 (mg/g) of total phenols and 2.75 (mg/g) of flavonoids. The concentrations of gallic acid, 3, 4-dihydroxybenzoic acid, chlorogenic acid and rutin in SPE were 0.05, 0.08, 0.08, and 0.51 (mg/g), respectively.

### Growth performance and carcass weight

The effects of the experimental treatments on the growth performance and hot and cold carcass weights of the lambs are shown in Table 3. There were no differences (p>0.05) among the treatments for DMI, ADG, FCR, BWG, the initial and final weights, and the hot and cold carcass weights of the lambs.

### The biochemical variables of plasma

The effects of the experimental treatments on biochemical variables of plasma are shown in Table 4. No differences (p> 0.05) were observed among the treatments for the biochemical variables of plasma, with the exception of cholesterol and TG concentrations (p<0.01 and p = 0.04, respectively). The lambs that received the ISPE and Vit E had lower (p< 0.01) plasma cholesterol concentration when compared with the control group. In addition, the plasma cholesterol concentration was also lower (p<0.01) in the lambs treated with the ISPE in comparison with the OSPE. No difference (p> 0.05) was observed between the control and other experimental groups for plasma TG, but the ISPE group showed lower (p<0.05) TG concentration than the OSPE and Vit E groups.

### Haematological variables

The effects of the experimental treatments on the haematological variables are presented in Table 5. There were no differences (p>0.05) among the experimental treatments for haematological parameters.

### Plasma antioxidant status and malondialdehyde level

The effects of the experimental treatments on the antioxidant status and MDA levels of plasma are presented in Table 6. There were differences (p<0.01) among the treatments for GPx activity of plasma. The lambs in the OSPE group had higher (p<0.01) plasma activity of GPx compared to the control group. In addition, GPx activity was also higher (p<0.01) in the OSPE group compared to the ISPE and Vit E groups.

Differences (p<0.01) were also observed among the treatments for the plasma activity of SOD. The lambs in the ISPE or Vit E groups had higher (p<0.01) plasma SOD activity than the control group. On the other hand, the Vit E group had greater (p<0.01) plasma SOD activity compared to the SPE groups. There were no differences (p>0.05) between the ISPE and the OSPE groups for plasma SOD.

No difference (p<0.05) was detected between the control group and other experimental groups for MDA levels of plasma. However, the ISPE group had lower (p<0.05) plasma levels of MDA than the OSPE group. Differences between the SPE groups and Vit E for the plasma level of MDA were non-significant. The plasma concentration of TAC was not affected (p>0.05) by the experimental treatments.

### Antioxidant status and malondialdehyde level in the tissues

The effects of the treatments on the antioxidant status and MDA levels of the longissimus dorsi muscle, liver, kidney and heart tissues are presented in Table 7. The experimental treatments had no effect (p>0.05) on GPx, SOD, TAC, and MDA levels of both the longissimus dorsi muscle and liver tissues. There were no differences (p>0.05) between the treatments for GPx activity of the kidney compared to the control, but GPx activity of the kidney was higher (p = 0.01) in the OSPE group compared to the ISPE group. The activity of GPx in the kidney tissue was similar among the SPE groups and Vit E. There were differences (p<0.01) among the treatments for GPx activity of the heart tissue. The OSPE group had higher (p<0.01) GPx activity of the heart compared to the control group. In addition, GPx activity of the heart tissue was also higher (p<0.01) in lambs treated in the OSPE group in comparison with either the ISPE or Vit E groups.

Differences (p<0.01) were also observed among the experimental treatments for SOD activity of the kidney tissue. SOD activity of the kidney was higher (p<0.01) in the OSPE and Vit E groups compared to the control group. In addition, SOD activity of the kidney in the OSPE group was also higher (p<0.01) than the ISPE group. No significant difference was observed between the SPE groups and Vit E for SOD activity of the kidney tissue. The activity of SOD in the heart tissue was not affected (p>0.05) by the experimental treatments.

The MDA levels of the kidney tissue were affected (p<0.01) by the experimental treatments. The kidney tissue levels of MDA in the experimental treatments were less (p<0.01) than the control group. There were no significant differences between other treatments for MDA levels of the kidney tissue. The level of MDA of the heart tissue in the Vit E group was lower (p<0.01) than the control group. However, no significant difference was detected between the ISPE and the OSPE groups for MDA levels of the heart tissue. The TAC in the tissues were not affected (p>0.05) by the experimental treatments.

## DISCUSSION

The total phenolic and flavonoid contents of SPE in this study were 4.29 (mg of GAE/g of powder extract) and 2.75 (mg QE/g of powder extract), respectively. Goli et al [8] and Termentzi and Kokkalou [28], respectively, reported total phenolic contents of 3.35 and 1.38 mg/g for Iranian saffron petal. Significantly greater amounts of total phenolic components (65.34 mg of GAE/g of powder extract) and flavonoids (60.64 mg CE/g of dry plant material) have been reported by Jadouali et al [29]. The reasons for the differences between our reported values and that of other researchers are probably attributed to the genetic variation, ecological conditions and/or the differences in the extraction method.

Flavonoids have been known to improve feed efficiency and growth rate in animals, likely through positive effects on metabolism as well as immune and antioxidant systems [30]. However, we failed to find any significant effects of SPE on the growth performance, probably due to low dose, few times and/or short duration of the trial. In agreement with our results, Omidi et al [31] reported that the use of ethanolic SPE in Baluchi lambs had no significant effect on BW. No effect of SPE on the growth performance of rats was also reported by Babaei et al [16]. However, these results are opposite to the results of Shirali et al [32] who found that the use of aqueous saffron extract in rats causes an increase in BWG. In the current study, the results relevant to vitamin E supported the findings of Maiorano et al [23], who intramuscularly injected a total dose of 1,500 IU of vitamin E into lambs and reported no influences on BW or hot and cold carcass weights. In another report, Zhao et al [33] showed that the use of vitamin E at concentrations of 20, 100, 200, and 2,000 IU had no effect on the initial weight, final weight, DMI, and FCR, but resulted in a significant reduction in ADG (over 100 IU d^−1^ lamb^−1^ of Vit E).

The evaluation of blood metabolites is important as an indicator of animal health. For example, increased liver enzymes (ALT, AST) in the blood indicate liver damage, or increased creatinine in the blood indicates kidney damage. In the current study, there was no effect of SPE and vitamin E on blood metabolites such as plasma levels of ALT and AST, TP, and creatinine, so it can be indicated that the applied doses of SPE had no negative impact on both the liver and kidney. The reduced cholesterol levels in this study could be attributed to the effects of SPE flavonoids on lipids metabolism [34]. Crocin as a bio-active compound in SPE [10] inhibits the activity of pancreatic lipase as a competitive inhibitor and plays an important role in reducing plasma cholesterol [35]. The effect of vitamin E to reduce plasma cholesterol level has previously been reported by Santos et al [36]. In this study, however, the plasma concentration of cholesterol and TG were significantly lower in the ISPE group compared to the OSPE group. This variety between the effect of the injectable and oral SPE on plasma cholesterol and TG may be associated to the changes of SPE compounds caused by both microbial and digestive enzyme activities through the gastrointestinal tract [37]. Omidi et al [31] reported that the oral administration of SPE (500–1,500 mg/kg BW) in lambs had no impact on blood metabolites. Also Ramadan et al [38], showed that the oral administration of ethanolic extracts of crocus sativus and propolis in a dose of 500 mg/kg of BW, and their half-dose combination, had no impact on plasma levels of AST, ALT, creatinine and urea in rats. The results related to vitamin E in this study are similar with findings of Vaswani et al [39], who reported that vitamin E supplementation had no significant impacts on ALT and AST activities in lambs.

In this study, no effect (p>0.05) of the experimental treatments was observed on the haematological parameters of lambs. Therefore, it seems that the oral and injection dosages of SPE had no anemic effects and were safe for the immune system of the lambs. Similar to our results, Alhidary and Abdelrahman [22] reported that the use of naringin supplementation in lambs at levels of 7 or 14 g/week had no effect on haematological variables. Babaei et al [16] also reported no effect (p>0.05) of SPE on haematology parameters in rats.

In this study, we observed no effect (p>0.05) of the SPE treatments (ISPE or OSPE) on the antioxidant status and MDA levels of both the longissimus dorsi muscle and liver. SOD and GPx activities of plasma, as well as GPx activity of the heart and SOD of the kidney, were significantly higher (p<0.01) in the SPE groups (ISPE and OSPE) compared to the control. In addition, the SPE treatments reduced (p<0.01) the level of MDA in the kidney tissue. Although reduced MDA concentration in the kidney could partially be attributed to the increased antioxidant enzymatic activity by SPE, a reason why SPE increased the antioxidant enzymatic activities can be related to the SPE flavonoids [40]. A literature has shown that plant polyphenolic flavonoids were one of the major groups of compounds acting as primary antioxidant free-radical terminators [41]. In addition to this, in our study, the activities of GPx and SOD of the plasma and tissues (kidney, heart) were higher (p<0.05) in the OSPE group than the ISPE group. We failed to find a clear reason to justify the variation between the injectable and oral administration of SPE. In a study, Omidi et al [31] reported that the oral administration of SPE elevated the plasma TAC without significant effect on the plasma MDA in lambs. In another report, Samarghandi et al [42] showed that aqueous saffron extract in a dose-dependent form (10, 20, and 40 mg/kg/d) in rats reduced serum MDA levels and significantly increased serum GPx and SOD activities.

Similar to the SPE treatments, the antioxidant status of the longissimus dorsi muscle and liver tissues was not affected by vitamin E. Salvatori et al [43] treated 15 day-old lambs by weekly intramuscular injection of 200 IU DL-α-tocopheryl acetate for 5 consecutive weeks and reported that muscle lipoperoxidation significantly reduced with Vit E treatment. Recently, Maiorano et al [23] reported higher lipid oxidative stability of meat in suckling male lambs treated weekly by intramuscular injection of 250 IU DL-α-tocopheryl acetate. The contradiction between our findings and the previous ones is likely due to the age of the lambs and/or to the site of injection (intramuscular vs subcutaneous). However, the activity of SOD in the plasma and kidney tissue of the Vit E lambs was greater (p<0.01) than the control group. In addition, the levels of MDA in both the kidney and heart tissues for Vit E were less (p<0.05) than the control group. It has been demonstrated that the relative bioavailability of Vit E varies between tissues [44]. This variation may partly associate to the capillary supply, which increases Vit E availability, and to the number of mitochondria in which the membrane-bound vitamin E accumulates [45]. The reason for the reduction of MDA levels of the kidney and heart with vitamin E can be related to the well-known antioxidant properties of Vit E in biological systems [46]. The Vit E prevents peroxidation of membrane lipids by scavenging lipid peroxyl radicals, and is changed into a tocopheroxyl radical as a consequence [47]. Ithayarasi and Devi [48] reported that α-tocopherol reduced lipid peroxidation in isoproterenol induced myocardial infarction in rats by maintaining the levels of antioxidants, membrane bound enzymes and activities of antioxidant enzymes near normal antioxidants, thus emphasizing the Vit E effects as an antioxidants. Similar to our finding for Vit E, Mokhber-Dezfouli et al [49] found that intramuscular Vit E injection had beneficial effects on plasma antioxidant activities, besides decreasing MDA values in newborn calves.

## CONCLUSION

Our results clearly indicated that the injectable SPE and Vit E decreased cholesterol levels in the plasma, but had no significant impacts on other blood metabolites, haematological parameters and growth performance of lambs. The antioxidant status and lipid oxidation in the muscle tissue and liver were not influenced by the experimental treatments. Also TAC levels in the plasma and tissues were not influenced by the experimental treatments. The oral administration of SPE significantly enhanced GPx activity in the plasma and heart tissue. The experimental treatments significantly decreased MDA levels in the kidney and heart tissues. These effects of the SPE were similar to those observed for the Vit E treatment. According to these findings, it could be concluded that SPE, in either oral or subcutaneous administration, had a potential to enhance the activity of antioxidant enzymes and to modulate lipids oxidation in lambs. Although the results achieved in this research revealed the appropriate effects of SPE and Vit E on the antioxidant statuses of Baluchi lambs, it is also recommended to improve the antioxidant status of other tissues, such as muscle and liver be used of SPE in higher doses, more often and/or for a longer trial period.

## Figures and Tables

**Table 1:** Ingredients and chemical composition of the basal diet (dry matter basis)

Ingredients (%)	
Barley, grain	33.08
Corn dry, grain	13.33
Soybean meal	12.75
Salt	0.42
Min Vit mixture	0.21
Limestone	0.21
Alfalfa hay	27.50
Wheat-straw	12.50
Chemical composition	
Dry matter (%)	88.32
Crude protein (%)	15.31
Non fiber carbohydrate (%)	44.91
Ether extract (%)	2.5
Neutral detergent fiber (%)	32.52
Acid detergent fiber (%)	21.60
Crude ash (%)	5.11
Metabolizable energy (Mcal/kg)	2.53

**Table 2:** Phenolic compounds and specific flavonoids detected in Iranian saffron petal extract using HPLC method

Compounds	Secondary metabolite survey1)
Total phenolic compounds (mg of GAE/g of powder extract)	4.29±0.15
Total flavonoids (mg QE/g of powder extract)	2.75±0.07
Galic acid (mg/g extract)	0.05± 0.01
3,4 DHB acid (mg/g extract)	0.08±0.02
Chlorogenic acid (mg/g extract)	0.08±0.01
Rutin (mg/g extract)	0.51±0.02

HPLC, high performance liquid chromatography; GAE, gallic acid equivalents; QE, quercetin equivalents; DHB, dihydroxybenzoic acid.

1) Data are presented as mean±standard deviation of three replicates.

**Table 3:** Effects of saffron petal extract and vitamin E on growth performances and carcass weight of Baluchi male lambs

Item	Treatments1)				SEM	p-value

	Control	ISPE	Vitamin E	OSPE		
DMI (kg/d)	1.56	1.50	1.44	1.78	0.04	0.06
Initial weight (kg)	34.38	35.30	35.14	36.06	0.39	0.98
Final weight (kg)	43.50	44.89	44.47.	47	0.32	0.80
ADG (g/d)	228.22	243.03	227.45	268.75	0.02	0.56
FCR	7.34	6.34	6.53	6.61	0.37	0.84
BWG (kg)	9.31	9.59	9.11	10.93	0.49	0.57
Hot carcass weight (kg)	17.53	16.96	17.80	18.63	0.72	0.87
Cold carcass weight (kg)	17.03	16.46	17.30	18.13	0.72	0.87

SPE, saffron petal extract; SEM, standard error of the mean; DMI, dry matter intake; ADG, average daily gain; FCR, feed conversion ratio; BWG, body weight gain; BW, body weight.

1) Control, 5 mL of physiological saline as a subcutaneous injection; ISPE, 25 mg/kg BW of SPE as a subcutaneous injection; Vitamin E, 225 IU of DL-α-tocopherol acetate as a subcutaneous injection; OSPE, 500 mg/kg BW of SPE as an oral dose.

**Table 4:** Effects of saffron petal extract and vitamin E on plasma metabolites of Baluchi lamb fed experimental diet.

Items	Treatments1)				SEM	p-value

	Control	ISPE	Vitamin E	OSPE		
Albumin (g/dL)	4.11	4.18	4.05	4.52	0.06	0.054
TP (g/dL)	6.89	7.47	7.21	8.19	0.41	0.77
ALT (U/L)	20.37	24.67	25.04	29.34	3.19	0.79
AST(U/L)	122.2	114.73	123.9	128.92	2.50	0.50
Creatinine (mg/dL)	0.96	1.14	1.05	2.59	0.50	0.72
TG (mg/dL)	18.51^ab^	17.10^b^	22.67^a^	23.42^a^	0.91	0.04
Cholesterol (mg/dL)	53.45^a^	41.92^b^	42.16^b^	57.33^a^	1.24	<0.01
Glucose (mg/dL)	81.14	83.95	80.56	84.59	1.49	0.79

SPE, saffron petal extract; SEM, standard error of the mean; TP, total protein; ALT, alanine aminotransferase; AST, aspartate aminotransferase; TG, triglyceride; BW, body weight.

1) Control, 5 mL of physiological saline as a subcutaneous injection; ISPE, 25 mg/kg BW of SPE as a subcutaneous injection; Vitaimin E, 225 IU of DL-α-tocopherol acetate as a subcutaneous injection; OSPE, 500 mg/kg BW of SPE as an oral dose.

a,b The means within the same row with at least one common letter, do not have significant difference (p>0.05).

**Table 5:** Effects of saffron petal extract and vitamin E on haematological variables of Baluchi lambs fed experimental diet

Items	Treatments1)				SEM	p-value

	Control	ISPE	Vitamin E	OSPE		
WBC (/μL)	13,789	11,490	13,017	9,653.86	568.70	0.12
N (/μL)	4,891.27	4,353.04	756,042	4,554.86	385.40	0.22
E (/μL)	249.53	155.86	268.67	245.85	21.31	0.32
L (/μL)	6,084.19	5,918.90	6,825.29	6,337.63	273.84	0.87
M (/μL)	232.27	185.67	318.06	337.18	17.07	0.39
RBC (×10^4^/μL)	10.76	10.44	10.66	10.40	0.07	0.62
HGB (g/dL)	11.47	11.09	11.29	11.14	0.06	0.39
PCV (%)	29.63	28.28	29.86	28.46	0.23	0.14
MCV (fL)	27.59	27.37	28.03	26.22	0.37	0.43
MCHC (g/dL)	38.54	39.18	38.06	39.32	0.19	0.09
Platelets (×10^5^/μL)	4.84	4.03	4.99	4.88	0.18	0.36

SPE, saffron petal extract; SEM, standard error of the mean; WBC, white blood cell count; N, neutrophilsp; E, eosinophil; L, lymphocytes; M, monocytes; RBC, red blood cell count; HGB, hemoglobin; PCV, hematocrit; MCV, mean cell volume; MCHC, mean corpuscular hemoglobin concentration; BW, body weight.

1) Control, 5 mL of physiological saline as a subcutaneous injection; ISPE, 25 mg/kg BW of SPE as a subcutaneous injection; Vitamin E, 225 IU of DL-α-tocopherol acetate as a subcutaneous injection; OSPE, 500 mg/kg BW of SPE as an oral dose.

**Table 6:** Effects of saffron petal extract and vitamin E on plasma antioxidant status of Baluchi lambs fed experimental diet.

Item	Treatments1)				SEM	p-value

	Control	ISPE	Vitamin E	OSPE		
GPx (U/L)	33.26^b^	39.35^b^	40.85^b^	53.49^a^	1.35	<0.01
SOD (U/L)	16.15^c^	27.16^b^	38.27^a^	21.87^bc^	0.91	<0.01
MDA(nmol/dL)	1.51^ab^	1.42^b^	1.62^ab^	1.87^a^	0.04	0.04
TAC (mmol/L)	0.28	0.29	0.31	0.30	0.008	0.64

SPE, saffron petal extract; SEM, standard error of the mean; GPx, glutathione peroxidase; SOD, superoxide dismutase; MAD, malondialdehyde; TAC, total antioxidant capacity; BW, body weight.

1) Control, 5 mL of physiological saline as a subcutaneous injection; ISPE, 25 mg/kg BW of SPE as a subcutaneous injection; Vitamin E, 225 IU of DL-α-tocopherol acetate as a subcutaneous injection; OSPE, 500 mg/kg BW of SPE as an oral dose.

a–c The means within the same row with at least one common letter, do not have significant difference (p>0.05).

**Table 7:** Effects of saffron petal extract and vitamin E on tissues antioxidant status of Baluchi male lambs

Items	Treatments1)				SEM	p-value

	Control	ISPE	Vitamin E	OSPE		
GPx (U/mg protein)						
Muscle	9.13	7.93	8.50	8.95	0.52	0.43
Liver	43.06	29.29	30.75	38.56	4.24	0.14
Kidney	32.32^ab^	29.28^b^	39.54^ab^	40.95^a^	1.15	0.01
Heart	28.36^b^	32.40^b^	30.51^b^	43.21^a^	1.71	<0.01
SOD (U/mg protein)						
Muscle	3.14	2.13	2.78	2.36	0.31	0.2
Liver	9.11	9.01	9.55	9.23	0.13	0.1
Kidney	4.22^c^	4.92^b^^c^	5.88^ab^	6.48^a^	0.16	<0.01
Heart	7.69	6.70	6.13	6.75	0.31	0.41
MDA (nmol/mg protein)						
Muscle	1.16	1.03	1.13	1.33	0.14	0.50
Liver	3.76	3.50	2.20	3.20	0.42	0.10
Kidney	3.25^a^	2.25^b^	2.16^b^	2.10^b^	0.07	<0.01
Heart	3.20^a^	3.00^a^	1.86^b^	2.50^ab^	0.10	<0.01
TAC (mmol/L)						
Muscle	0.07	0.10	0.13	0.08	0.02	0.40
Liver	1.02	1.14	1.27	1.06	0.08	0.31
Kidney	0.16	0.18	0.15	0.20	0.016	0.20
Heart	0.075	0.09	0.11	0.076	0.02	0.65

SPE, saffron petal extract; SEM, standard error of the mean; GPx, glutathione peroxidase; SOD, superoxide dismutase; MAD, malondialdehyde; TAC, total antioxidant capacity; BW, body weight.

1) Control, 5 mL of physiological saline as a subcutaneous injection; ISPE, 25 mg/kg BW of SPE as a subcutaneous injection; Vitamin E, 225 IU of DL-α-tocopherol acetate as a subcutaneous injection; OSPE, 500 mg/kg BW of SPE as an oral dose.

a,b The means within the same row with at least one common letter, do not have significant difference (p>0.05).
